# Supplementary material for: Needle Tract Seeding after Endoscopic Ultrasound Tissue Acquisition of Pancreatic Lesions: A Systematic Review and Meta-Analysis
Source: Diagnostics (Basel). 2022 Aug 31;12(9):2113. doi: 10.3390/diagnostics12092113 (PMC9498098; doi:10.3390/diagnostics12092113)

ONLINE SUPPLEMENT

Supplementary Table S1. Risk of bias assessment and quality of included studies.

	Observational studies[a]			
	**Selection**	**Comparability**	**Outcome**	**Overall quality**
Kitano 2022	***	NA	**	H
Park 2022	**	*	**	M
Ngamruengphong 2013	***	**	**	H
Micames 2003	**	*	*	L
Yoon 2014	**	*	*	H
Ikezawa 2013	**	*	*	L
Maruta 2021	**	*	**	M
Nakatsubo 2021	**	NA	*	L
Suzuki 2018	**	*	**	M
Tsutsumi 2016	***	**	**	H

L, low; H, high; U, unclear; M, moderate.

[a] Study quality assessment performed by means of Newcastle/Ottawa scale (each asterisk represents if the respective criterion within the subsection was satisfied)

Supplementary Figure S1. Leave-one-out analysis of pooled incidence of needle tract seeding after exclusion of the study by Kitano et al

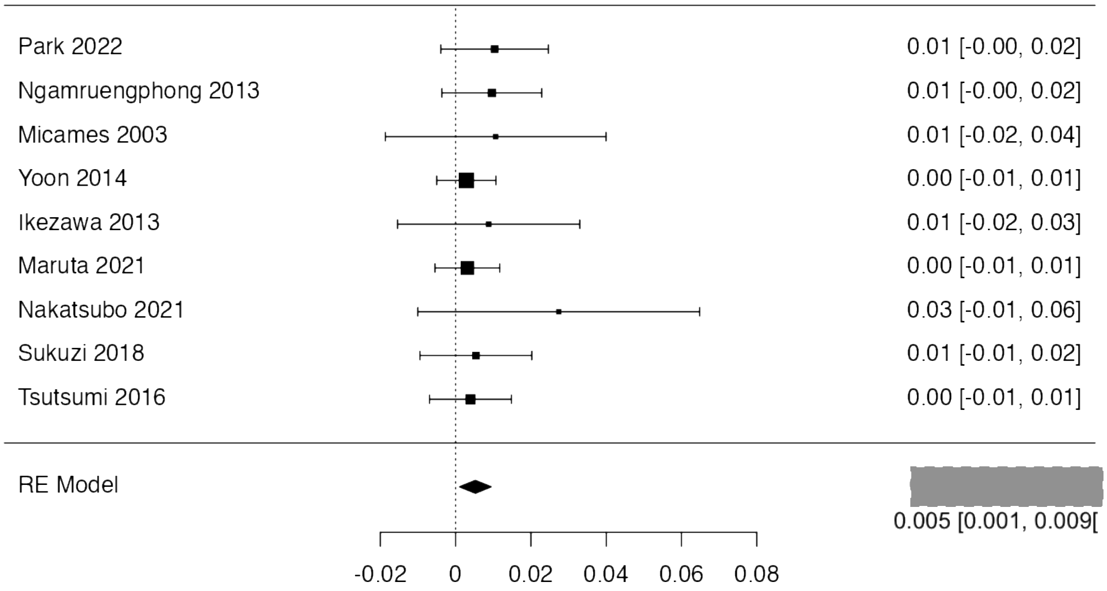

Supplementary Figure S2. Pooled rates of needle tract seeding in patients with pancreatic adenocarcinoma

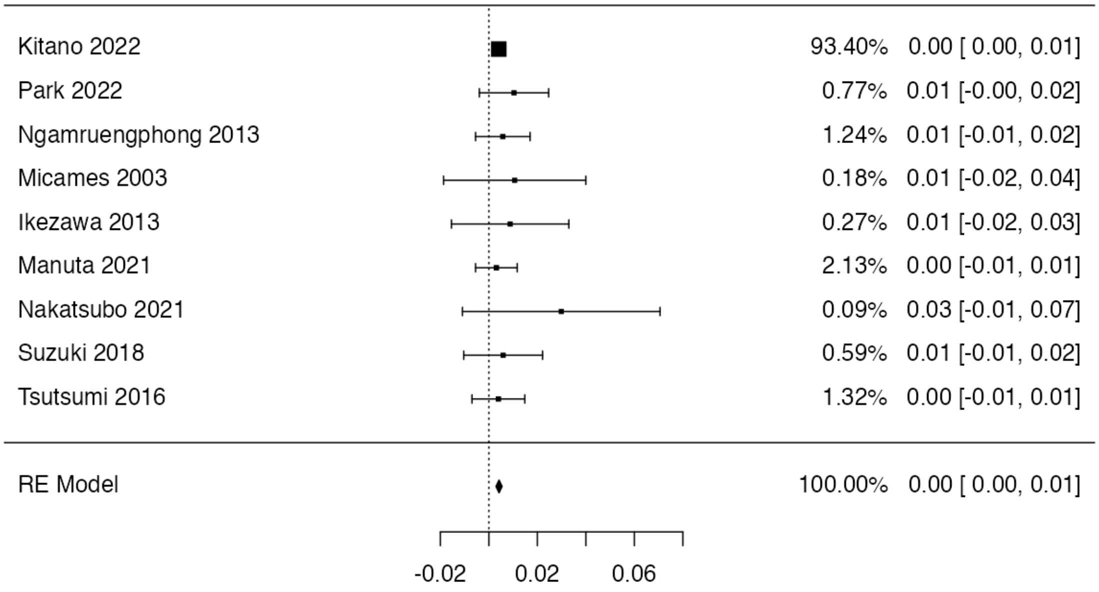

Supplementary Figure S3. Pooled rates of needle tract seeding in patients with pancreatic cysts

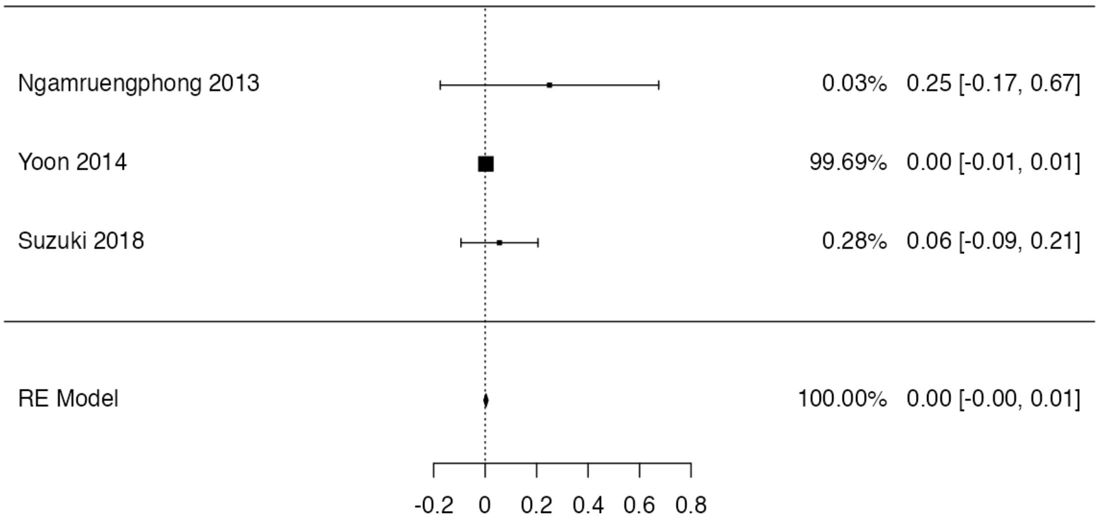

Supplementary Figure S4. Funnel plot concerning the comparison between EUS-FNA vs non-FNA in terms of peritoneal dissemination

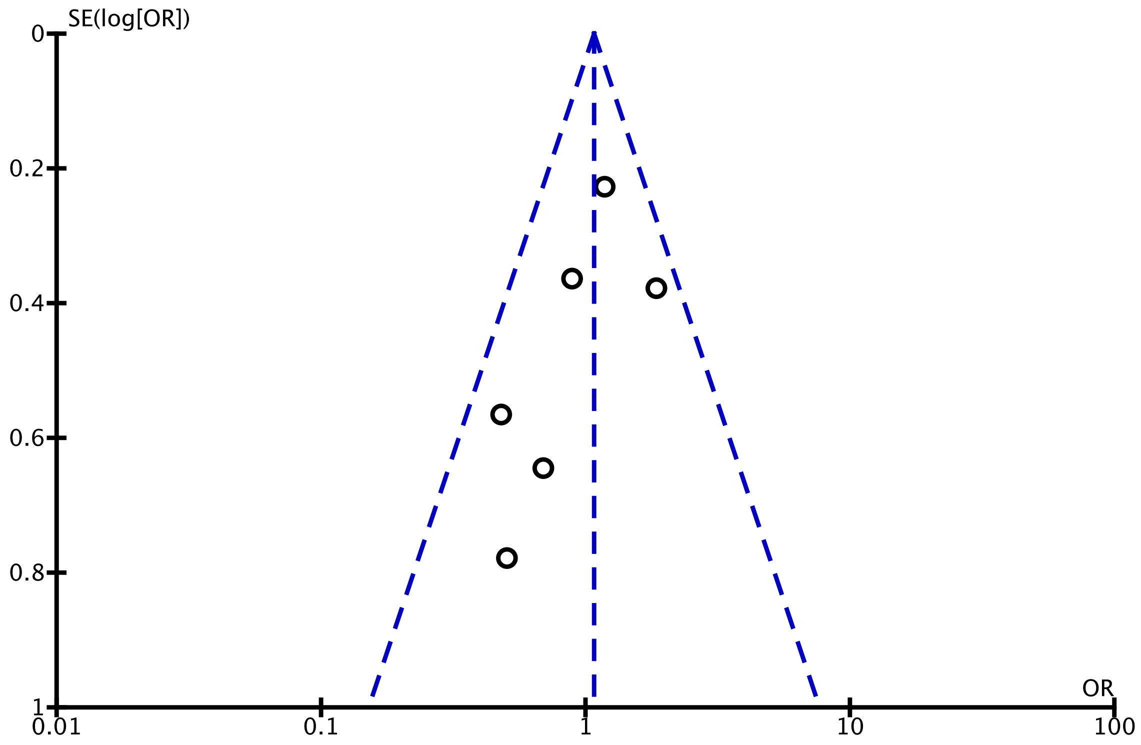

Supplement: Supplementary file 1 [file diagnostics-12-02113-s001.zip › diagnostics-1888353-supplementary.pdf]
